# Marsupialization and peripheral ostectomy for the management of large odontogenic keratocyst: a case report

**DOI:** 10.1093/jscr/rjad119

**Published:** 2023-03-17

**Authors:** Ali Khalil, Ziad Albash, Nadim Sleman, Wadie Sayegh

**Affiliations:** Department of Oral and Maxillofacial Surgery, Faculty of Dentistry, Tishreen University, Latakia, Syria; Department of Oral and Maxillofacial Surgery, Faculty of Dentistry, Tishreen University, Latakia, Syria; Department of Oral and Maxillofacial Surgery, Faculty of Dentistry, Tishreen University, Latakia, Syria; Department of Oral and Maxillofacial Surgery, Faculty of Dentistry, Tishreen University, Latakia, Syria

## Abstract

Odontogenic keratocyst has been of particular interest due to its distinctive behavior and its tendency to frequently recurrence and the diversity of treatment methods. Researchers have differed over the past decades about the nature of this lesion, sometimes it was classified as a cyst and sometimes it was classified as a tumor because of its specific histopathologic features, high recurrence rate and aggressive behavior. We discuss a case of a large odontogenic keratocyst (OKC) that was treated by marsupialization followed by peripheral ostectomy. Based on our findings, we conclude that the marsupialization followed by peripheral ostectomy was a conservative and effective option for the management of large OKC.

## INTRODUCTION

The first description of odontogenic keratocyst (OKC) was published in 1956 by Philipsen. The lesion has been of particular interest because of its specific histopathologic features, high recurrence rate and aggressive behavior [1].

This lesion was excluded in 2005 from the WHO classification of the odontogenic cysts, and it was classified as a neoplasm and was called keratocystic odontogenic tumor (KCOT) [2]. In January 2017, the tumor was backed into the cyst category as OKC according to the 4th edition of the World Health Organization’s Classification of Head and Neck Tumors [3].

Several options are available for the treatment of OKC depending on several factors. These options include: enucleation, curettage, resection and marsupialization. The resection and enucleation are considered invasive surgical procedures, but they are the most common treatments [4]. The marsupialization and decompression are more conservative surgical techniques, and may be suitable options.

The marsupialization was first described by Partsch for the cystic lesions of the jaws [5]. The principle of this technique is the externalization of the cyst. This can be done via the making of a window in the mucosa and the cystic liner, and suturing them to make an opened cavity that communicates with the oral cavity [5]. The aim of the marsupialization and the decompression is to reduce the size of the cystic lesion by reducing the pressure to stimulate new bone formation.

Peripheral ostectomy is a technique that involves enucleation of the lesion with peripheral bone removal using rotary instruments [6].

We discuss a case of a large OKC that was treated by marsupialization followed by peripheral ostectomy.

## CASE PRESENTATION

**Figure 1:**
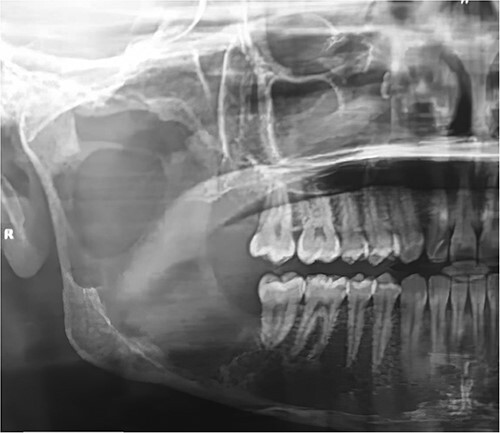
OPG showed radiolucent lesion including most of the right ramus area.

**Figure 2:**
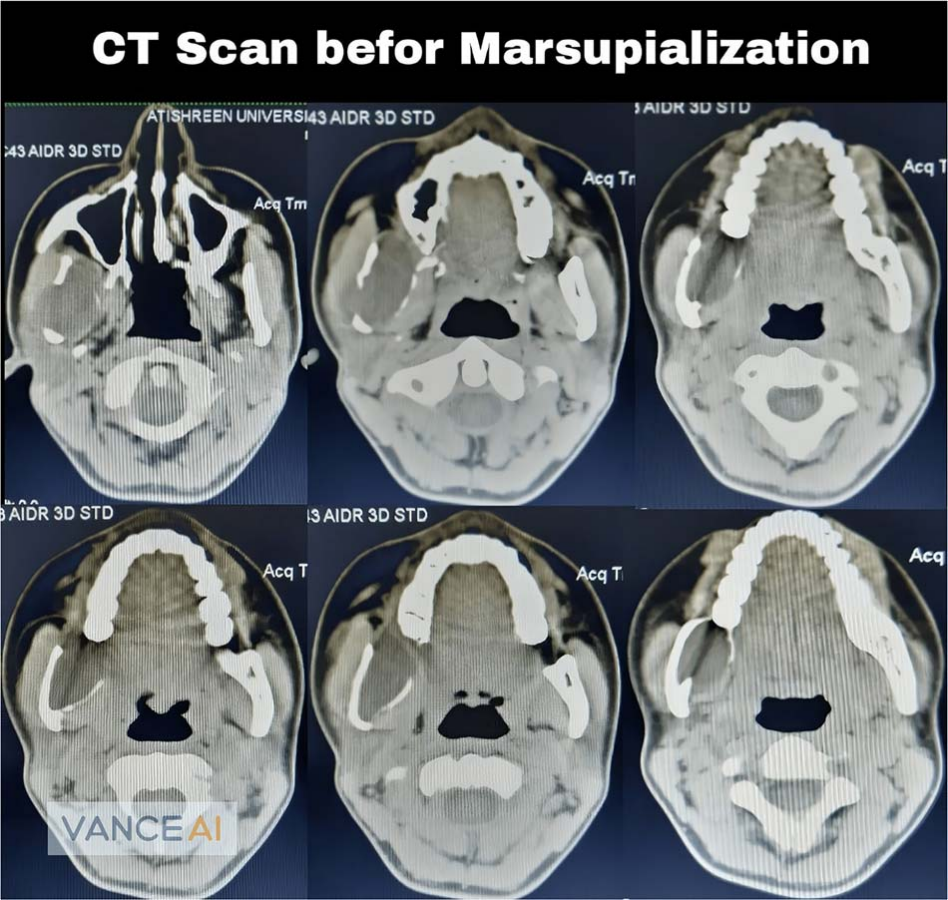
Computed tomography scan showed a cavity in the right mandibular ramus with expansion and absence of most buccal and lingual bone plates.

A 22-year-old male patient presented to the Oral and Maxillofacial Surgery Department at Tishreen University Hospital with chief complaints, recurrent fistula at the right retromolar area and clear yellow liquid discharge intraorally from the fistula 1 year ago. The patient was recommended an Orthopantomogram (OPG) which showed radiolucent lesion, including most of the right ramus area (Fig. 1). Computed tomography (CT) scan showed a cavity in the right mandibular ramus with an expansion and absence of most buccal and lingual bone plates (Fig. 2). An incisional biopsy was performed and a specimen was sent for histopathology examination. Based on clinical, radiographical and histological features, the lesion was diagnosed as an OKC.

**Figure 3:**
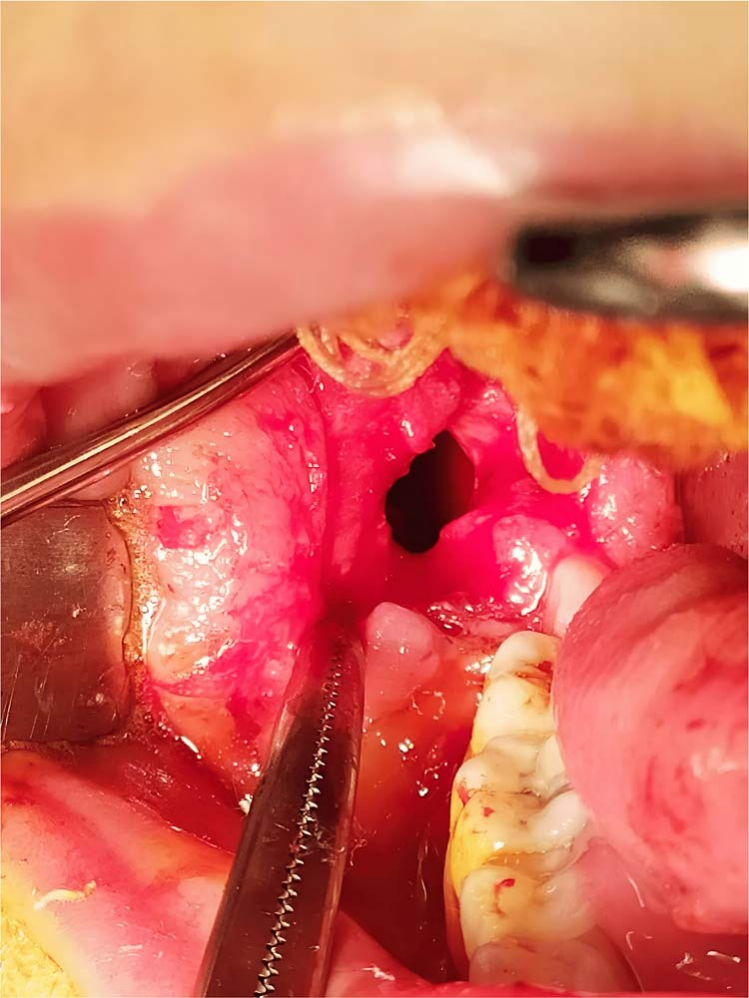
Marsupialization procedure: exposure of the cystic cavity, 1 cm size window was made into the cyst cavity.

**Figure 4:**
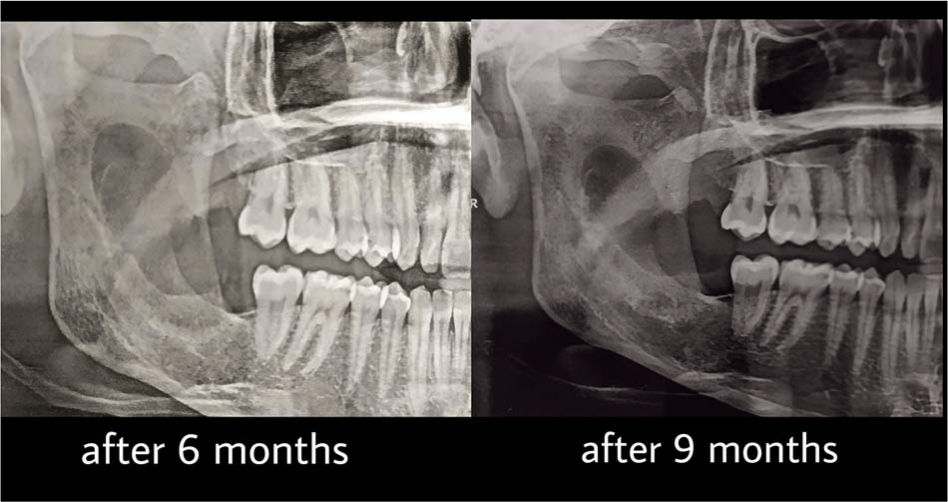
Follow-up after 6 and 9 months.

**Figure 5:**
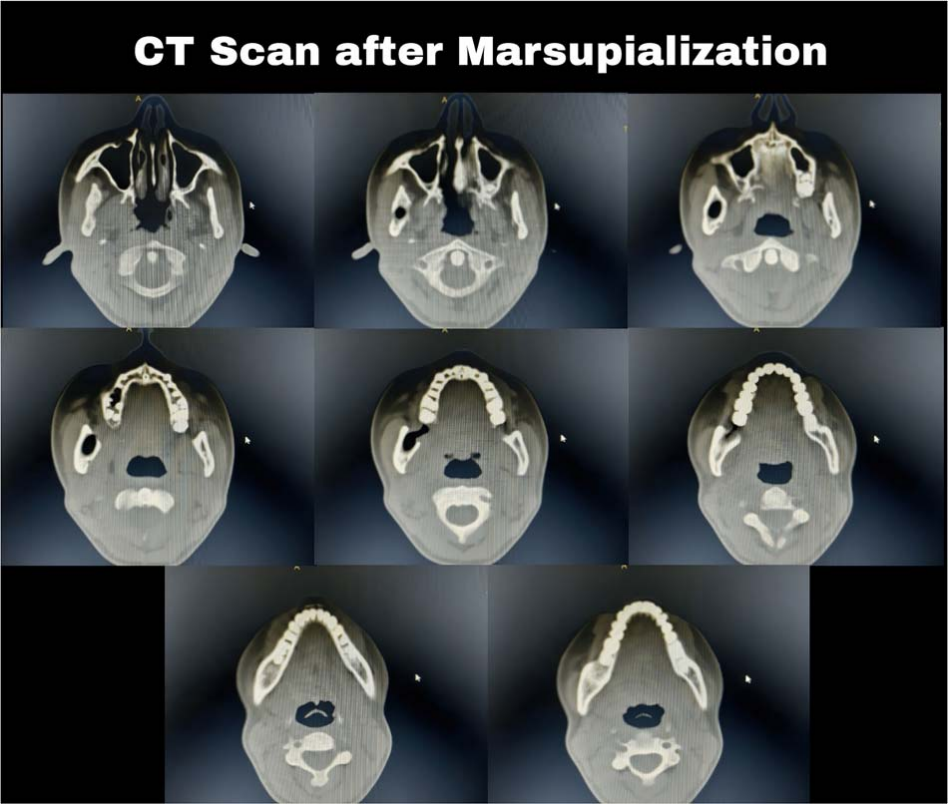
Computed tomography after 12 months showed a bone regeneration in the lesion’s area and the cystic lesion has become completely surrounded by thick bony plates.

Under general anesthesia, an incision was made around the fistula to remove the overlying mucosa and expose the lesion. A 1 cm size window was made into the cyst cavity (Fig. 3). The cyst lining was sutured to surrounding mucosa. The cavity was kept open using a povidone iodine-saturated gauze, and it was replaced every 2 weeks. Follow-up radiograph (OPG) was recommended after 3, 6, 9 and 12 months (Figs 4 and 5).

**Figure 6:**
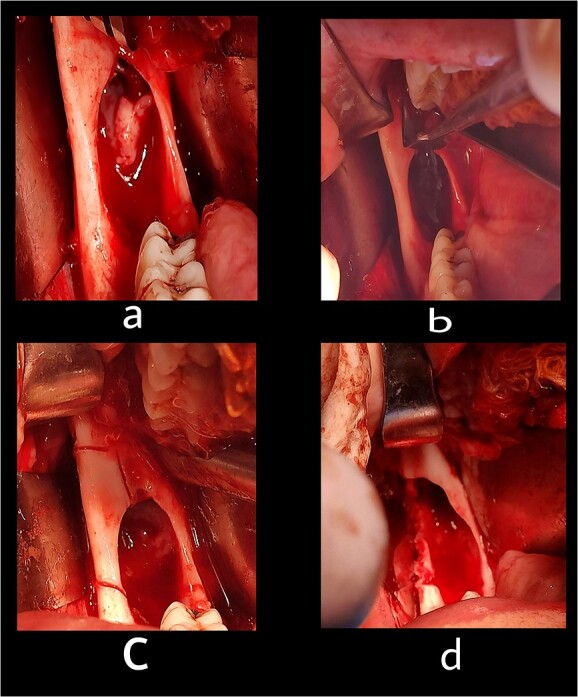
The second stage: (**a**) surgical exposure of the right anterior border of the ramus. (**b**) The cystic cavity after the enucleation of the lesion. (**c**) Removal of the buccal plate to allow access to the entire of the lesion. (**d**) The cystic cavity after removal of buccal plate and peripheral ostectomy.

**Figure 7:**
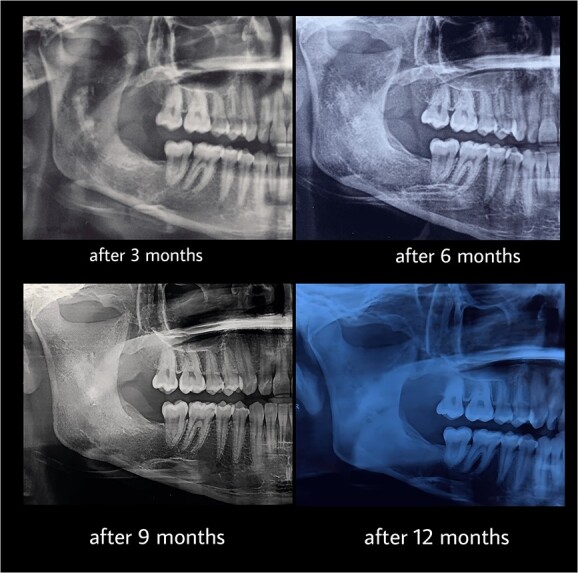
Follow-up after 3, 6, 9, 12 months of the second stage.

**Figure 8:**
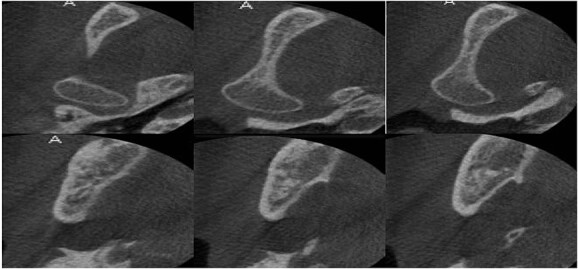
Cone beam computed tomography after 30 months showed full healing without any recurrence.

After 12 months, the second stage was performed to remove the lesion (Fig. 6). Under general anesthesia, enucleation with peripheral ostectomy was performed. A flap was developed to expose the anterior border and most of the lateral surface of ramus. The lining of the lesion was detached from the bony walls. A surgical bur was used to remove bone adjacent to the cystic lining. After enucleation, the cavity was filled with gelfoam and the incisions were closed with 4–0 Prolene suture. The patient returned regularly for follow-up after every 3 months (Fig. 7). The total follow-up period so far was 36 months. Cone beam computed tomography (CBCT) scan after 24 and 36 months showed no evidence of lesion recurrence and great bone formation (Fig. 8).

## DISCUSSION

A major shift occurred in our understanding and management of keratinous cysts in 2005 when they were classified as tumors**.** However, reclassification of these lesions as odontogenic cysts in 2017 is due to new evidence regarding their behavior and histological composition especially that they tend to regress after decompression.

In recent years, many scientific papers have been published on the efficacy of the marsupialization for the treatment of various types of cystic lesions in the oral region [7, 8], and even cystic ameloblastoma [9]. The marsupialization was described as a less aggressive option because it maintains the developing dentition, reduces cyst size [7, 9] minimizes the damage of the important anatomical structures (inferior alveolar nerve and sinus) and it stimulates osteogenesis [9].

Several studies have shown that the rate of recurrence of keratinized cysts is the highest when the lesion is managed by enucleation alone, but it is the most common surgical option for the treatment of OKCs in literature [10]. The lowest recurrence rate of keratinized cysts in literature was after resection (ranging from 1.85 to 2.2%) [11]. Although it is associated with the lowest recurrence rate, it is the most aggressive procedure, that causes many postoperative complications, and requires a specific plan for reconstruction.

Marsupialization are currently considered as non-definitive therapies in KCOT treatment by the majority of authors due to the odontogenic epithelium remains in the cyst cavity [12]. Marsupialization requires complementary therapies that include: simple enucleation, radical enucleation, enucleation with Carnoy’s solution, enucleation and cryotherapy and enucleation with peripheral ostectomy. The combination of enucleation with adjunctive techniques reduces high recurrence rates that present with simple enucleation and optimizes the treatment [7, 8].

Simple enucleation differs from enucleation with peripheral ostectomy in that the enucleation with peripheral ostectomy is followed by the removal of 1.5–2 mm of bone with a handpiece in KCOT margins [6]. Several studies reported that the combination of enucleation [13] with peripheral ostectomy demonstrated positive results, with low recurrence rates [13–15]. A retrospective review in 2005 found that enucleation followed by peripheral ostectomy was associated to a decrease in recurrence rates. In 2007, Kolokythas *et al*. [14] reported a recurrence rate of 0% with enucleation followed by peripheral ostectomy.

## CONCLUSION

Based on our findings with the limitations of our case report, we concluded that the marsupialization followed by peripheral ostectomy was a conservative and effective option for the treatment of large OKC. No postoperative complications and no recurrence were observed after 36 months.

## Data Availability

All corresponding data used to support the findings of this study are included within the article.
